# From workplace to home environment: spreading of mouse allergens by laboratory animal workers

**DOI:** 10.1007/s00420-020-01603-9

**Published:** 2020-11-21

**Authors:** Hannah Kube, Ronald Herrera, Gisela Dietrich-Gümperlein, Rudolf Schierl, Dennis Nowak, Katja Radon, Laura Wengenroth, Jessica Gerlich

**Affiliations:** 1 Institute and Clinic for Occupational, Social and Environmental Medicine, University Hospital, LMU Munich, Munich, Germany; 2 Comprehensive Pneumology Center (CPC) Munich, German Center for Lung Research (DZL), Munich, Germany

**Keywords:** Laboratory mice, Mouse allergens, Mus m1 allergen, Allergy

## Abstract

**Purpose:**

Laboratory animal workers (LAW) working with laboratory mice are exposed to mouse allergens (MA). If MA are spread to home environments, this might increase the risk for allergies in LAW and their families. This study aimed to assess 1. whether spreading of MA from workplace to home environment takes place; 2. which factors increase spreading of MA.

**Methods:**

In a cross-sectional study, dust samples were taken on the mattress and seating in homes of LAW (*n* = 105) and an unexposed comparison group (*n* = 13). From 89 LAW, additional dust samples were taken from their workplaces. Samples were analysed using Mus m1 ELISA kits [detection limit (DL) 0.2 ng mus m1/ml]. Sociodemographic data, personal history of allergies and cleaning habits, as well as work-related characteristics (LAW only) were assessed by questionnaire. Latent factors were assessed via factor analysis. Tobit models were fitted to analyse the latent factors’ contribution to MA spreading.

**Results:**

MA concentration on the seating was significantly higher in home environments of LAW (median = 1.28 ng mus m1/m^2^) than in the comparison group (median < DL, *p* = 0.019). The highest workplace MA concentration was found on the floor of the scullery (median = 140,000.00 ng mus m1/m^2^), followed by hair-covering caps (median = 76.02 ng mus m1/m^2^). Cage and mouse facility cleaning tasks and infrequent changing of bed linen at home were statistically significantly associated with higher MA concentrations at home.

**Conclusions:**

Spreading of MA from LAW’s workplace to their home environment takes place, especially among LAWs involved in cleaning tasks.

**Electronic supplementary material:**

The online version of this article (10.1007/s00420-020-01603-9) contains supplementary material, which is available to authorized users.

## Introduction

In Germany, mice constitute the biggest proportion of laboratory animals (Bundesministerum für Ernährung und Landwirtschaft (BMEL) 2018). Therefore, many German laboratory animal workers (LAW) are potentially exposed to urinary protein Mus m1, which due to its small size is easily spread in the workplace (Ohman et al. 1994). Exposure to Mus m1 may lead to sensitization among LAW in a dose-dependent manner (Matsui et al. 2004). For LAW working with small animals, the annual incidence for occupational rhinitis is 2.54/1000 LAW and for occupational asthma 1.56/1000 LAW (Draper et al. 2003). To the best of our knowledge, the specific incidence for allergies in LAW exposed to mice is unknown. Prevalence of laboratory animal allergy in cross-sectional studies in various countries ranged from 6 to 44 percent (Corradi et al. 2012).

To prevent workers from developing occupational allergies and asthma, various preventive measures have been implemented, aiming at reducing exposure levels at the workplace. Nevertheless, the resulting reduction in prevalence and incidence of occupational allergies and asthma was only small (Folletti et al. 2008). One reason for this could be a transfer of allergens from the workplace to the home environment, which would increase the duration of exposure for the LAW (Simoneti et al. 2016). Such spreading of allergen exposure was shown e.g. for farmers and an intervention to reduce allergen spreading from the barn to the home was shown to reduce the fraction of exhaled nitric oxide [FE(NO)] over time, indicating that airway inflammation decreased in farmers with occupational asthma (Dressel et al. 2007, 2009; Radon et al. 2000).

There is first evidence that also a Mus m1 transfer from the workplace to LAW’s homes takes place (Krop et al. 2007). This study from the Netherlands, including 15 LAW and 15 controls, indicated that uncovered hair might be the most important risk factor for the allergen transfer from the workplace to LAW’s mattresses. An early report indicated that indirect exposure to Mus m1 can cause sensitization even among children of LAW (Krakowiak et al. 1999). So far, no other studies have analysed the potential spreading of allergens from the laboratory to the home environment of LAW, nor were the risk factors contributing to exposure levels at home studied.

Therefore, this study aimed to assess 1. whether spreading of mice allergens (MA) Mus m1 from workplace to home environment takes place in German animal facilities; 2. which work-related and person-related factors are associated with the MA concentration found in home environments.

## Materials and methods

### Study design and participants

In a cross-sectional study, dust samples were taken from the homes of LAW working with laboratory mice, mouse tissues and body fluids. Additional dust samples were taken from their workplaces in animal facilities. To control for MA originating from other sources than from laboratory mice (*Mus musculus forma domestica*) such as house mice (*Mus musculus domesticus*) or field mice (*Mus musculus musculus*), a small comparison group not working with mice and not keeping pet mice was also included.

Heads of animal facilities in Munich were involved in the planning phase of the study in order to review practical aspects of the study. Study recruitment was accomplished via a national mailing list of heads of German animal facilities. Once the head of an animal facility indicated his interest in the study, the study team presented the study on-site and invited LAW to participate in the study. Furthermore, students with laboratory mouse contact were invited through a mailing list of medical student communities and graduate schools in Munich. The comparison group was recruited in the private surroundings of the authors, including persons with and without pets who were not occupationally exposed to mice. The candidates for the comparison group were chosen as a convenience sample, where response calculation was not possible.

### Assessment of mouse allergen concentration

Dust samples were taken in the homes of all study participants. Two localizations were sampled: the seating where the study participants first sat down when they got home from work and the part of the mattress where head and upper body were lain down. The top bed sheet was removed before taking the mattress sample.

For each LAW, additional samples were taken if possible at the workplace from the locker, seating, offices, sculleries, changing rooms and staff rooms (if existent). In addition to the surface samples at the workplaces, hair-covering caps that were worn during work were collected afterwards in jars with screw caps for analysis. These samples served the purpose of determining the MA load during working time (Krop et al. 2007).

Surface dust samples were collected using a commercial vacuum cleaner “Bosch BSG62030 Bodenstaubsauger 2000W LOGO” according to a standardized procedure based on studies with German farmers (Berger et al. 2005; Radon et al. 2000). The vacuum cleaner was equipped with a collector and a convenient filter [DUSTREAM^®^ (40 micron nylon mesh), Indoor Biotechnologies, Ltd, Vision Court, Caxton Place, Cardiff CF23 8HA UK]. For each sample, a total surface of one square metre was vacuumed for two minutes with maximum power, moving the suction tube of the vacuum cleaner at a 45° angle across the surface. Areas smaller than one square metre were recorded. The dust samples were stored in sealable plastic tubes at room temperature. Upon arrival in the laboratory, all samples were extracted in phosphate buffered saline containing 0.05% Tween 20 with a pH value of 7.4 (PBS-T) on a vibrating plate for two hours. Volume depended on MA sample size. The extracts were centrifuged at 2500 rpm for 20 min, carefully transferred in separate tubes and stored at  − 20 °C. Extracts were analysed using a sandwich ELISA with biotin and streptavidin detection Mus m1 ELISA kit (EL-MM1, Indoor Biotechnologies). Mus m1 ELISA uses a sensitive detection of the species *Mus musculus* with a 0.2 nanogram Mus m1/ml detection limit (DL). MA concentrations were expressed as nanogram per square meter (m^2^). Samples smaller than 1m^2^ were standardized for 1 m^2^.

### Assessment of explanatory variables

Through a questionnaire, socio-demographic data like age, gender and animal-related profession (in LAW) and level of education (in the comparison group) were assessed. Additional topics such as personal history of allergies, keeping pets and cleaning habits that could affect allergen exposure or spreading of MA were included (Krop et al. 2007). In LAW, also work conditions, work tasks, types of cages used, use of protective clothing and protective behaviour were assessed. Questions considering allergies were taken from the European Community Respiratory Health Survey (Burney et al. 1994). Questions considering profession, keeping pets and cleaning habits were adapted from the SOLAR study and the Lower Saxony Lung Study (Heinrich et al. 2011; Radon and Schulze 2006). Work-related questions were developed with the heads of animal facilities in Munich and pilot tested with some LAW. Participants could decide whether to complete a digital or a paper version of the questionnaire.

Paper questionnaire and sampling protocol data was entered twice in EpiData to assure accuracy.

### Statistics

Stata version 12 and R were used for analyses (Heinrich et al. 2011; R Core Team 2017; Radon and Schulze 2006). Comparisons between LAW and the comparison group and within LAW were performed using Kruskal–Wallis test. *P* values below 0.05 were considered statistically significant. In LAW, factor analysis was used to account for mutual correlations among variables describing tasks at work, clothing in animal house, hygienic behaviour after work and private conditions/behaviour. Factor analysis was applied separately to these four groups of variables and missing data was imputed (Supplemental Tables 1–4b).

Because some samples’ MA concentration was below the detection limit, Tobit models were fitted using the R package VGAM to study the association between the MA levels at home and covariates (Breen 1996; Yee 2010). The Tobit model is a special case of the more general censored regression model, and it has been used in multiple applications in epidemiology (Alvear Rodriguez and Tovar Cuevas 2018; Arostegui et al. 2012; Garcia-Esquinas et al. 2013) especially in environmental epidemiology (Harnly et al. 2009; Lubin et al. 2004; Sarnat et al. 2006). Using this approach, a linear regression is applied to uncensored continuous data, and this regression is conditioned to an assumed influence on censored data. Regression coefficients in Tobit models must be interpreted as the linear association on the uncensored latent variable and not on the observed variable (Breen 1996).

Given the small sample size in this study, Tobit–Bayesian models were estimated as a sensitivity analysis using the R library MCMCpack (Chib 1992; Martin et al. 2011). Tobit–Bayesian models were estimated using Markov Chain Monte Carlo (MCMC) with a Gibbs sampler. Multivariate Gaussian priors were used on the coefficients of the covariates, and an Inverse Gamma prior for the conditional error variance. 30,000 MCMC iterations were used with a burn-in number of 1000. The thinning interval was set to 10.

## Results

### Characteristics of the study population

The study population included 105 LAW from 17 animal facilities and 13 members of the comparison group who all filled in the questionnaire and provided dust samples from their homes. More females (75% in LAW, 92% in the comparison group) than males participated in the study (Table 1).

**Table 1:** Characteristics of LAW and comparison group

	LAW (*n* = 105)		Comparison group (*n* = 13)	
	*n*	%	*n*	%
Sociodemographic characteristics				
Gender				
Female	79	75	12	92
Male	26	25	1	8
Age				
(Mean/SD)	Mean = 35	SD = 10	Mean = 43	SD = 15
Education				
No qualification for university entrance	n.a	n.a	1	8
Qualification for university entrance	n.a	n.a	12	92
Animal attendant	50	48	n.a	n.a
Laboratory assistant	10	10	n.a	n.a
University	44	42	n.a	n.a
Missing	1			
Health status				
Asthma confirmed by physician				
No	89	86	11	85
Yes	15	14	2	15
Missing	1			
Any type of allergy confirmed by physician				
No	74	71	10	77
Yes	30	29	3	23
Missing	1			
Parental asthma, allergy, or eczema				
No	51	61	7	58
Yes, one parent	28	34	5	42
Yes, both parents	4	5	0	0
Do not know or missing	22		1	

Mean age was 35 years in LAW and 43 years in the comparison group. LAW had predominantly trained as animal attendants (48%) or had a university degree (42%). Most members of the comparison group were qualified for university entrance (92%). Asthma was prevalent in 14% of LAW and in 15% of the comparison group, while allergies were prevalent in 29% of LAW and in 23% of the comparison group. Most LAW had direct mouse contact at work and were also involved in cleaning tasks (71%, *n* = 74). Sixty-four percent (*n* = 67) of LAW operated with open cages.

### MA concentration at home

For logistic reasons in LAW, 56 study participants (53%) had to take the samples at home themselves. All samples for the comparison group were taken by the study team. The median MA concentrations at homes of the comparison group were lower than at homes of LAW. Differences reached statistical significance for MA concentrations on the seating (Table 2).

**Table 2:** MA concentration at home in LAW and comparison group

Sampling place	MA concentration^a^ in LAW (*n* = 105)				MA concentration^a^ in comparison group (*n* = 13)					*p *value^b^
	Median	Min	Max	*n* < DL	Median	Min	Max	*n* < DL		
Seating	1.28	< DL	235.88	35	< DL	< DL	98.55	10	0.019	
Mattress	2.68	< DL	106.91	28	0.39	< DL	87.94	6	0.055	

*DL* detection limit

^a^Mouse allergen concentration measured in mus m1 ng/m^2^

^b^*p* value from chi-squared probability using non-parametric Kruskal–Wallis test

### MA concentration at work

Workplace samples could be taken for 89 LAW. The highest MA concentration at work was found on the floor of sculleries (median = 140,000.00 mus m1 ng/m^2^), followed by hair-covering caps (median = 76.02 mus m1 ng/m^2^) and the changing room floors (median = 66.33 mus m1 ng/m^2^; Table 3). In all rooms, MA concentrations were highest on the floors compared to chairs, lockers or shelves.

**Table 3:** MA concentration at work for LAW

Sampling place	MA concentration^a^				
	Median	Min	Max	n < DL	Number of samples taken^b^
Offices					
Floors	9.68	< DL	2703.00	5	30
Chairs	5.24	< DL	8202.81	3	36
Changing rooms					
Lockers	28.52	< DL	3105.43	10	65
Shelves	23.67	< DL	755.20	5	21
Floors	66.33	1.03	224,900.00	0	27
Staff rooms					
Floors	10.23	< DL	6929.50	1	17
Chairs	6.60	< DL	82,099.09	2	18
Laboratories					
Floors	6.99	< DL	24,425.00	1	15
Chairs	4.13	< DL	335.58	4	16
Scullery^c^					
Floors	140,000.00	14,096.00	150,000.00	0	3
Hair-covering caps	76.02	0.54	12,302.00	0	82

*DL* detection limit

^a^Mouse allergen concentration measured in mus m1 ng/m^2^

^b^Numbers of samples taken vary due to how locations were accessed by LAW. If several LAW used chairs, lockers or floors together, we only took one sample of each place accessed

^c^In the scullery the mouse cages are emptied from bedding and litter and are then cleaned

### Home-related factors associated with MA concentration at home

Study participants who changed their linen less than once a month had a higher MA concentration on their mattress (median = 3.20 mus m1 ng/m^2^) than those who changed them more frequently (median = 0.65 mus m1 ng/m^2^; *p* = 0.025), while age of the mattress was not associated with MA concentration. Education, frequency of vacuuming, keeping pets, using public transport, participants’ storey and house type were also not associated with MA concentration at home.

Whether the MA sample at home was taken by the study team or by the participant was not associated with the MA concentration found in homes.

### Work-related factors associated with MA concentration at home

The MA concentrations at home were higher in LAW who fulfilled cleaning tasks at work than in those who did not, reaching statistical significance for the seating (median = 2.11 mus m1 ng/m^2^ in LAW fulfilling cleaning tasks vs. median < DL, *p* = 0.042). Hours per month working with mice, total years worked with mice and type of cages used were not associated with MA concentrations at home. Likewise, wearing full protective clothing (hair-covering cap, mouth protection, gloves, coat) in the animal facility was not associated with the MA concentrations at home.

Hygiene practices after work (none, changing clothes, taking air shower, taking shower) were also not associated with MA concentrations at home. However, those who showered had a higher MA concentration on their hair-covering cap before taking the shower (median = 110.12 mus m1 ng/m^2^) than those who changed their clothes and/or took an air shower (median = 87.37 mus m1 ng/m^2^) and those who did none of these procedures after work (median = 11.65 mus m1 ng/m^2^; *p* < 0.001).

### Combination of home and work-related factors

Factor analysis in LAW revealed work-related factors (cleaning mouse facilities, cleaning cages, handling mice in the laboratory), factors related to clothing (head and face protection, whole body protection) and factors related to hygienic behaviour after work (taking shower while still at work, taking shower at home; Supplemental Tables 5a–8b).

All of those factors included at least two covariables with factors loadings > 0.5 and were included in a univariate Tobit model. No meaningful factors were found for private conditions and behaviour; hence, the according single variables were directly included in the subsequent models.

In the univariate Tobit models, living with a household member who also worked with mice significantly increased MA concentration on the seating at home [Beta = 49.61, 95% confidence interval (20.32; 78.98), Table 4]. The work-related factors “cleaning mouse facilities” and “cleaning cages “ showed statistically significant associations with an increase in MA concentration on the mattress [5.25, (0.99; 9.51); 5.13 (0.77; 9.48) respectively]. Moreover, less frequent bed linen changing was statistically significantly associated with higher MA concentrations on the mattress [8.54 (1.10; 15.98)]. Statistically significant associations in the adjusted model were “cleaning mouse facilities” and “cleaning cages”, which were associated with a statistically significant increase in MA concentration on the mattress [4.14, (0.03; 8.26); 4.96, (0.84; 9.08)]. Moreover, changing bed linen less than once a month was associated with a higher MA concentration on the mattress as well [8.79, (1.64; 15.93)].

**Table 4:** Tobit regression models for the outcomes MA concentrations on the seating and mattress

Factors/variables	MA concentration on seating						MA concentration on mattress						
	Model 0 (unadjusted)			Model 1 (adjusted)			Model 0 (unadjusted)			Model 1 (adjusted)			
	Beta	95% CI	*p* value	Beta	95% CI	*p* value	Beta	95% CI	*p* value	Beta	95% CI		*p *value
Factor W1: cleaning mouse facilities	− 1.19	(− 12.43; 10.04)	0.835				**5.25**	**(0.99; 9.51)**	**0.016**	**4.14**	**(0.03; 8.26)**		**0.048**
Factor W2: cleaning cages	7.22	(− 4.28; 18.72)	0.218				**5.13**	**(0.77; 9.48)**	**0.021**	**4.96**	**(0.84; 9.08)**		**0.018**
Factor W3: handling mice in laboratory	− 4.65	(− 16.85; 7.55)	0.455				2.43	(− 2.15; 7.01)	0.298				
Factor C1: head and face protection	− 5.36	(− 16.31; 5.60)	0.338				0.37	(− 4.09; 4.84)	0.870				
Factor C2: whole body protection	− 3.38	(− 15.97; 9.20)	0.598				1.65	(− 3.05; 6.34)	0.492				
Factor H1: taking shower at work	− 5.97	(− 17.43; 5.49)	0.307				0.35	(− 3.87; 4.58)	0.869				
Factor H2: taking shower at home	− 2.32	(− 13.99; 9.35)	0.697				1.27	(− 3.18; 5.72)	0.576				
Age (continuous)	0.54	(− 0.43; 1.51)	0.274				− 0.25	(− 0.64; 0.14)	0.206				
Sex (female vs. male)	3.94	(− 19.36; 27.23)	0.740				4.70	(− 4.14; 13.53)	0.298				
Keeping pets at home (yes vs. no)	− 2.20	(− 21.85; 17.45)	0.826				3.67	(− 3.92; 11.26)	0.344				
Additional household members (1 vs. 0)	22.55	(− 3.56; 48.66)	0.091	11.04	(− 14.96; 37.05)	0.405	7.69	(− 2.20; 17.59)	0.128				
Additional household members (2 vs. 0)	8.16	(− 18.26; 34.57)	0.545	3.57	(− 21.86; 29.01)	0.783	1.29	(− 8.79; 11.37)	0.802				
Other household member working with mice (yes vs. no)	**49.65**	**(20.32; 78.98)**	**0.001**	**44.70**	**(14.28; 75.12)**	**0.004**	8.78	(− 2.90; 20.47)	0.141				
Vacuuming (weekly vs. daily)	17.89	(− 13.44; 49.21)	0.263				5.49	(− 6.13; 17.11)	0.355				
Vacuuming (≤ biweekly vs. daily)	26.60	(− 10.72; 63.93)	0.162				1.50	(− 12.86; 15.85)	0.838				
Bed linen (≤ 1/month vs. > 1/month)^a^							**8.54**	**(1.10; 15.98)**	**0.024**	**8.79**	**(1.64; 15.93)**		**0.016**
Mattress age (> 3 years vs. ≤ 3 years)^a^							3.87	(− 3.97; 11.70)	0.333				
Regular stable visits (yes vs. no)	− 10.17	(− 0,43; 0,23)	0.550				5.24	(− 7.20; 17.67)	0.409				

Results in bold indicate significance (*p* < 0.05)

*MA* mouse allergen concentration, *CI* confidence interval

^a^Not included in the regression models for the outcome MA concentration on seating

## Discussion

The results of this study showed that MA were widely spread inside German laboratory animal facilities and to a lower extent also in LAW’s home environments. The MA concentration on the seating at home was significantly higher in LAW than in the comparison group not working with mice. Thus, it could be shown for the first time that spreading of MA from workplace to the home environment takes place in German laboratory animal facilities.

Both home-related and work-related factors influenced the MA concentration at home. Living with a household member who also worked with mice significantly increased MA concentration on the seating at home. Changing bed linen less than once a month as well as cleaning mouse facilities and mouse cages were associated with higher MA concentrations on the mattress. Mouse cages are usually cleaned by emptying them in the scullery, where very high MA concentrations were found, in accordance with other studies (Feistenauer et al. 2014). Animal facilities were commonly cleaned using brooms, mops or vacuum cleaners. During dry cleaning, dust stirs up and thus also MA, which might then settle on LAW’s skin and clothes. These results were in line with the expectations based on previous recommendations. Wet cleaning is recommended in contrast to sweeping the floor (Corradi et al. 2012; Harrison 2001; Stave and Darcey 2012; Thulin et al. 2002).

MA were also found in the comparison groups’ homes, which might be explained by the suspected role of MA as a common environmental allergen. Other studies also found MA in home environments, e.g., in more than 95% of US-American inner-city households (Matsui et al. 2005; Phipatanakul et al. 2000), on 70% of living room floors in New Zealand and in 46% of inner-city households in Poland (Peters et al. 2006; Stelmach et al. 2002). Krop et al. found significantly higher MA concentrations on the mattress of 6 LAW than in 15 controls (Krop et al. 2007). A trend for higher MA concentrations on the mattress in LAW than in the comparison group was seen here as well, but results were not statistically significant. In contrast to the results by Krop et al., MA concentrations on mattresses were not associated with mattress age in the presented study. This might be explained by the older age of mattresses in Krop’s study (range 1–20 years) where more MA might have accumulated compared to the study presented here (range 0–12 years). The study by Krop et al. indicated that keeping cats or dogs (which might have contact with MA outdoors) as pets was associated with a higher concentration of MA and rat urinary allergens in the mattress, which could not be confirmed here. Again, this might be explained by the older mattresses in Krop’s study.

In accordance with Krop et al., no association between number of working hours and MA concentration at home was found in this study (Krop et al. 2007). The study presented here also indicates that there is no association between time worked with mice (hours in the last four weeks and total years) and the MA concentration at home. Other studies showed that the type of cages used affected the MA concentration in the mouse rooms (Feistenauer et al. 2014; Gordon et al. 1997; Renstrom et al. 2001). However, considering MA concentrations at home, the presented study did not find an association between cage type and MA concentrations at home. A possible explanation could be that LAW adjust their hygienic behaviour after work to the MA concentrations they are exposed to, e.g., in this study, LAW who were exposed to high MA concentrations during work were more likely to take a shower directly after work.

### Limitations and strengths

Unfortunately, we cannot estimate response in our study. Information about the study was distributed via different channels, resulting in a convenience sample and making it impossible to count the number of recipients who received an invitation for participation. Female participants constituted a big part of the study population. However, this setting is probably rather representative of the LAW population in Germany, given the high proportion of female workers in animal handling work in Germany (Statistisches Bundesamt 2014). Some of the MA samples weighed far less than one gram; therefore, ng Mus m1 per m^2^ was reported instead of ng Mus m1 per gram. Standard methods for measuring MA concentrations do not exist, which makes it unadvisable to compare the measured values with those of other studies. Mus m1 was the only assessed MA in this study; however, it is considered suitable for the evaluation of MA concentrations and suggested for exposure level assessment (Ferrari et al. 2004). Study participants fulfilled very heterogeneous work tasks in various locations of the respective animal facility. Thus, it was not possible to assign a certain type of MA work place sample as potential continuous factor explaining MA concentration at home. Instead, specific work tasks were included in models explaining MA concentration at home. Our study was not intended to elucidate the effect of low-level MA exposure at home. Thus, this aspect will have to be investigated in further studies. Furthermore, speculating whether low-level home exposure may be suitable for primary allergy prevention in children living in such households is far beyond the scope of our study.

It can be considered a strength of the study that the heterogeneity of work tasks and protective behaviour were considered and adjustments for many potential confounders like keeping pets, house type or usage of public transport were made. Furthermore, samples were taken at various places at home and at the workplace, allowing a multifaceted tracking of MA concentrations. Another major strength of the study presented here is the incorporation of a comparison group, facilitating to control for MA as a common environmental allergen. However, a larger comparison group would have allowed to further distinguish MA concentration in persons of different socio-economic status and life styles. Finally, the results of the study were disseminated so that each participant received their personal MA concentration measurements and each participating animal facility was informed about the anonymized measured values. This direct dissemination of results allows evaluating levels of MA exposure and defining hot spots of increased MA concentration, and can thus help to decrease the exposure to MA both for LAW and their families.

## Conclusions

This study showed for the first time that MA were widely spread inside German laboratory animal facilities and to a lower extent also in LAW’s home environments. Cleaning cages and cleaning mouse facilities were the most important work-related factors contributing to higher MA concentrations at home. Additionally, MA concentrations rose if another household member worked with mice as well and with infrequent changing of bed linen.

Special focus should be given to a reduction of MA concentration at work during cleaning tasks and especially in the sculleries, where the MA concentration was at its highest. Finally yet importantly, changing bed linen more frequently than once a month is a recommendable measure to reduce exposure in the home environment. Households with more than one LAW should pay special attention to reduce MA concentration at home.

However, it is known from the literature that the risk for sensitization to rat allergen is not linearly associated with increasing exposure (Cullinan et al. 1999; Jeal et al. 2006). Authors found an increasing risk of sensitization with increasing exposure, but at high exposure levels the risk decreased again. One reason for these phenomena might be a survival bias leading to a highly exposed LAW cohort where only LAW without allergic symptoms remain. Another explanation might be a high-dose tolerance among those LAW who are highly exposed. However, it is still unknown at which threshold MA concentration and dose starts to become clinically relevant. Further studies in different countries are needed to evaluate the impact of the recommended interventions to take into account for international differences in the structure and arrangements of mouse facilities. Moreover, future research should also be carried out on sensitization levels for MA.

## Electronic supplementary material

Below is the link to the electronic supplementary material.

Supplementary file1 (DOCX 33 KB)
