# Supplementary material for: From workplace to home environment: spreading of mouse allergens by laboratory animal workers
Source: Int Arch Occup Environ Health. 2020 Nov 21;94(4):601–10. doi: 10.1007/s00420-020-01603-9 (PMC8068679; doi:10.1007/s00420-020-01603-9)
Supplement: Supplementary file 1 — Supplementary file1 (DOCX 33 KB) [file 420_2020_1603_MOESM1_ESM.docx]

**Supplemental table 1. Variables tested for factor analysis: Tasks last 4 weeks**

| **Tasks last 4 weeks** | **No** | | | | **Yes** | | | | **Missing** |
| --- | --- | --- | --- | --- | --- | --- | --- | --- | --- |
|  | **n** | | **%** | | **n** | | **%** | | **n** |
| Cage change | 33 | | 34 | | 65 | | 65 | | 1 |
| Wean pubs | 43 | | 44 | | 55 | | 56 | | 1 |
| Weigh mice | 65 | | 66 | | 33 | | 34 | | 1 |
| Inject mice | 57 | | 58 | | 41 | | 42 | | 1 |
| Shave mice | 85 | | 87 | | 13 | | 13 | | 1 |
| Surgery | 77 | | 79 | | 21 | | 21 | | 1 |
| Retrieve organs | 39 | | 40 | | 59 | | 60 | | 1 |
| Handle tissue/blood samples | 62 | | 63 | | 36 | | 37 | | 1 |
| Stack dirty cages | 28 | | 29 | | 70 | | 71 | | 1 |
| Empty dirty cages | 64 | | 65 | | 34 | | 35 | | 1 |
| Set up clean cages | 67 | | 68 | | 31 | | 32 | | 1 |
| Other tasks | 75 | | 77 | | 23 | | 23 | | 1 |
|  | **No** | | | **Yes, wet** | | **Yes, dry** | | | **Missing** |
|  | **n** | **%** | | **n** | **%** | **n** | | **%** | **n** |
| Cleaning tasks | 43 |  | | 21 |  | 34 | |  | 1 |

**Supplemental table 2. Variables tested for factor analysis: Clothing in animal house**

| **Clothing in animal house** | **No** | | **Yes** | | **Missing** |
| --- | --- | --- | --- | --- | --- |
|  | **n** | **%** | **n** | **%** | **n** |
| Scrubs | 17 | 17 | 82 | 83 | 0 |
| One-way overall | 71 | 72 | 28 | 28 | 0 |
| Cotton overall | 83 | 84 | 16 | 16 | 0 |
| Gloves | 1 | 1 | 98 | 99 | 0 |
| Surgical mask | 17 | 17 | 82 | 83 | 0 |
| FFP2 mask | 84 | 85 | 15 | 15 | 0 |
| Head cap | 11 | 11 | 88 | 89 | 0 |
| hood | 80 | 81 | 19 | 19 | 0 |
| Other | 67 | 68 | 32 | 32 | 0 |

**Supplemental table 3. Variables tested for factor analysis: Hygienic behaviour after work**

| **Hygienic behaviour after work** | **No** | | **Yes** | | **Missing** |
| --- | --- | --- | --- | --- | --- |
|  | **n** | **%** | **n** | **%** | **n** |
| *At workplace* |  |  |  |  |  |
| Shower | 81 | 83 | 17 | 17 | 1 |
| Washing hair | 85 | 87 | 13 | 13 | 1 |
| Air shower | 87 | 89 | 11 | 11 | 1 |
| Change clothes | 45 | 46 | 53 | 54 | 1 |
| *At home* |  |  |  |  |  |
| Shower | 36 | 37 | 62 | 63 | 1 |
| Washing hair | 46 | 47 | 52 | 53 | 1 |
| Change clothes | 32 | 33 | 66 | 67 | 1 |
|  | **At home** | | **At work** | | **Missing** |
|  | **n** | **%** | **n** | **%** | **n** |
| Washing work clothes | 10 | 10 | 88 | 90 | 1 |

**Supplemental table 4.a Variables tested for factor analysis: Private animal contact**

| **Private animal contact** | **No** | | **Yes** | | **missing** |
| --- | --- | --- | --- | --- | --- |
|  | **n** | **%** | **n** | **%** | **n** |
| Pets at home | 48 | 48 | 51 | 52 | 0 |
| Private visits to animal stables last 5 yrs. | 66 | 72 | 26 | 28 | 7 |
| Household member with regular mouse contact | 87 | 89 | 11 | 11 | 1 |

**Supplemental table 4.b Variables tested for factor analysis: Conditions at home**

| **Conditions at home** | **n** | **%** | **missing (n)** |
| --- | --- | --- | --- |
| Age sleeping mattress |  |  | 2 |
| 0 – 3 yrs. | 57 | 59 |  |
| >3 yrs. | 40 | 41 |  |
| Frequency changing bedlinen |  |  | 1 |
| >1 per month | 50 | 51 |  |
| ≤1 per month | 48 | 49 |  |
| Frequency vacuuming |  |  | 1 |
| Daily | 13 | 13 |  |
| Weekly | 69 | 70 |  |
| Every other week or fewer | 16 | 16 |  |
| Number of additional household members |  |  | 2 |
| 0 | 22 | 23 |  |
| 1 | 38 | 39 |  |
| ≥2 | 37 | 38 |  |

**Supplemental table 5a. Rotated factor analyses: Tasks last 4 weeks**

| **Factor** | **Variance** | **Proportion** | **Cumulative** |
| --- | --- | --- | --- |
| Factor 1 | 2.26 | 0.41 | 0.41 |
| Factor 2 | 1.51 | 0.28 | 0.69 |
| Factor 3 | 1.45 | 0.26 | 0.95 |
| Factor 4 | 0.52 | 0.10 | 1.05 |
| Factor 5 | 0.35 | 0.06 | 1.11 |
| Factor 6 | 0.27 | 0.05 | 1.16 |

**Supplemental table 5b. Rotated factor loadings: Tasks last 4 weeks**

| **Variable** | **Factor W1** | **Factor W2** | **Factor W3** | **Factor W4** | **Factor W5** | **Factor W6** |
| --- | --- | --- | --- | --- | --- | --- |
| **Cage change** | **0.82** | 0.09 | 0.03 | -0.09 | 0.05 | -0.04 |
| **Wean pubs** | **0.73** | 0.02 | -0.11 | 0.09 | 0.02 | 0.11 |
| Weigh mice | 0.00 | -0.20 | 0.05 | 0.04 | 0.39 | -0.05 |
| **Inject mice** | -0.05 | 0.00 | **0.51** | 0.09 | 0.28 | -0.08 |
| **Shave mice** | 0.12 | -0.03 | **0.55** | -0.05 | -0.12 | -0.20 |
| Surgery | -0.09 | -0.03 | 0.76 | 0.14 | 0.03 | 0.03 |
| Retrieve organs | -0.02 | -0.20 | 0.34 | 0.33 | 0.06 | -0.07 |
| Handle tissue/blood samples | -0.26 | -0.09 | 0.39 | 0.44 | 0.11 | -0.08 |
| **Cleaning tasks** | **0.66** | 0.28 | -0.06 | -0.14 | -0.18 | 0.21 |
| **Stack dirty cages** | **0.66** | 0.32 | 0.12 | -0.37 | -0.04 | -0.04 |
| **Empty dirty cages** | 0.13 | **0.80** | -0.04 | -0.09 | -0.16 | 0.02 |
| **Set up clean cages** | 0.27 | **0.77** | -0.07 | -0.09 | -0.15 | 0.13 |
| Other tasks | 0.06 | 0.05 | -0.08 | -0.03 | -0.04 | 0.37 |

**Supplemental table 6a. Rotated factor analyses: Clothing in animal house**

| **Factor** | **Variance** | **Proportion** | **Cumulative** |
| --- | --- | --- | --- |
| Factor 1 | 1.41 | 0.47 | 0.47 |
| Factor 2 | 1.08 | 0.36 | 0.82 |
| Factor 3 | 0.68 | 0.23 | 1.05 |
| Factor 4 | 0.33 | 0.11 | 1.16 |
| Factor 5 | 0.19 | 0.06 | 1.22 |

**Supplemental table 6b. Rotated factor loadings: Clothing in animal house**

| **Variable** | **Factor C1** | **Factor C2** | **Factor C3** | **Factor C4** | **Factor C5** |
| --- | --- | --- | --- | --- | --- |
| Scrubs | 0.23 | -0.12 | 0.54 | -0.08 | -0.09 |
| One-way overall | -0.02 | 0.73 | 0.10 | -0.01 | 0.00 |
| Cotton overall | -0.04 | -0.06 | -0.55 | 0.03 | -0.05 |
| Gloves | 0.02 | 0.06 | -0.03 | 0.47 | 0.10 |
| Surgical mask | 0.79 | -0.09 | 0.13 | 0.38 | 0.09 |
| FFP2 mask | -0.17 | 0.06 | 0.08 | -0.48 | 0.18 |
| Head cap | 0.84 | -0.11 | 0.20 | 0.00 | 0.02 |
| hood | -0.14 | 0.71 | -0.13 | 0.04 | 0.03 |
| other | 0.03 | 0.01 | -0.01 | 0.00 | 0.40 |

**Supplemental table 7a. Factor analyses: Hygienic behaviour after work**

| **Factor** | **Variance** | **Proportion** | **Cumulative** |
| --- | --- | --- | --- |
| Factor 1 | 1.62 | 0.50 | 0.50 |
| Factor 2 | 1.46 | 0.45 | 0.95 |
| Factor 3 | 0.48 | 0.15 | 1.10 |
| Factor 4 | 0.24 | 0.07 | 1.17 |

**Supplemental table 7b. Factor loadings: Hygienic behaviour after work**

| **Variable** | **Factor H1** | **Factor H2** | **Factor H3** | **Factor H4** |
| --- | --- | --- | --- | --- |
| *At work* |  |  |  |  |
| Wash clothes | 0.14 | 0.08 | 0.29 | 0.25 |
| Shower | 0.87 | -0.15 | 0.18 | -0.04 |
| Wash hair | 0.86 | -0.11 | 0.15 | -0.12 |
| Air shower | -0.08 | 0.03 | -0.05 | 0.36 |
| Change clothes | 0.12 | 0.03 | 0.45 | -0.10 |
| *At home* |  |  |  |  |
| Shower | -0.24 | 0.79 | 0.23 | 0.12 |
| Wash hair | -0.10 | 0.80 | 0.15 | 0.09 |
| Change clothes | 0.02 | 0.40 | -0.22 | -0.02 |

**Supplemental table 8a. Rotated factor analyses: Private conditions**

| **Factor** | **Variance** | **Proportion** | **Cumulative** |
| --- | --- | --- | --- |
| Factor 1 | 0.81 | 0.92 | 0.92 |
| Factor 2 | 0.50 | 0.56 | 1.48 |
| Factor 3 | 0.19 | 0.21 | 1.69 |

**Supplemental table 8b. Rotated factor loadings: Private conditions**

| **Variable** | **Factor P1** | **Factor P2** | **Factor P3** |
| --- | --- | --- | --- |
| Other household members | -0.47 | 0.02 | 0.11 |
| Age mattress | -0.01 | 0.01 | 0.25 |
| Frequency changing bedlinen | 0.39 | 0.22 | 0.28 |
| Frequency vacuuming | 0.60 | -0.10 | 0.10 |
| Pets at home | -0.19 | 0.40 | 0.02 |
| Private visits to animal stables | 0.22 | 0.37 | 0.17 |
| Other household members with mouse contact | 0.00 | 0.37 | -0.02 |
